# Critical vitamin D and iron intakes in infants aged 6–11 months: results from the nationwide German KiESEL study

**DOI:** 10.3389/fnut.2025.1472685

**Published:** 2025-02-17

**Authors:** Leonie Burgard, Clarissa Spiegler, Sara Jansen, Anna-Kristin Brettschneider, Andrea Straßburg, Ute Alexy, Stefan Storcksdieck genannt Bonsmann, Regina Ensenauer, Thorsten Heuer

**Affiliations:** ^1^Department of Nutritional Behaviour, Max Rubner-Institut (MRI) – Federal Research Institute of Nutrition and Food, Karlsruhe, Germany; ^2^Department of Child Nutrition, Max Rubner-Institut (MRI) – Federal Research Institute of Nutrition and Food, Karlsruhe, Germany; ^3^Department of Nutritional Epidemiology, Institute of Nutrition and Food Sciences, University of Bonn, Bonn, Germany

**Keywords:** nutrient intake, vitamin D, infants, commercial infant foods, nutrition survey, Germany

## Abstract

**Background:**

Nutrient intake during the phase of complementary feeding is pivotal for healthy development, yet current data for European infants are lacking.

**Objective:**

To provide latest data on energy and nutrient intake of infants in Germany, to compare these with the Dietary Reference Values (DRVs) of the European Food Safety Authority, and to assess the contribution of vitamin D supplementation and commercial infant foods to nutrient intake.

**Design:**

Analyses are based on weighed food records (3 + 1 day) of 118 infants aged 6–11 months from the representative cross-sectional Children’s Nutrition Survey to Record Food Consumption (KiESEL) conducted in Germany from 2014 to 2017. Energy and nutrient intake was calculated using the German Nutrient Database BLS 3.02, the LEBTAB database, and a supplement database.

**Results:**

Median energy and protein intakes were above DRVs, while fat intake was below. Dietary vitamin and mineral intakes mostly met or exceeded the DRVs. However, iron intake reached neither the Population Reference Intake nor the Average Requirement. Vitamin D intake from foods was below half the Adequate Intake (AI). When additionally considering vitamin D supplements, administered to 51.7% of infants, the AI was reached. Girls’ iodine intake was below the DRVs. In contrast, the intakes found for retinol equivalents, vitamin K, and vitamin C were about four times the DRVs. Commercial infant foods were key dietary sources for all nutrients for which intakes deviated considerably from DRVs.

**Conclusion:**

Micronutrient intake, particularly of iron and vitamin D, needs improving in infants aged 6–11 months in Germany. Vitamin D supplementation was a prerequisite for meeting the AI, confirming the necessity for vitamin D supplements in infancy and the promotion of the respective recommendations. The considerable up- and downward deviations from DRVs point to a need for adjusting fortification levels of commercial infant foods in European legislation.

## 1 Introduction

Nutrition in early life is crucial for optimal development and health throughout the course of life (1). At about 6 months of age, infants’ energy and nutrient needs gradually exceed the amounts provided by human milk, requiring the addition of complementary foods (2). During this sensitive stage of life, nutrient requirements are high relative to body weight (3) and thus can be challenging to meet. As stated in an opinion paper by the European Food Safety Authority (EFSA) in 2013, vitamin D, iron, and in some countries iodine are considered as potentially critical micronutrients in European infants (4). For vitamin D, this is addressed by the recommendation for routine supplementation in infants (5).

Despite the given concerns, nationally representative data of infants’ nutrient intake in Europe are limited, with only few publications covering the period of complementary feeding (6–9). In Germany, the latest published data including infants stem from a survey conducted in 2001–2002 (10). Since then, infant diets have most likely changed in response to the ever-transforming market of commercial complementary foods (11) and changing trends in feeding practices (12), therefore requiring new data specific to this vulnerable age group. A secondary analysis of food record data from the European Childhood Obesity Project (CHOP) cohort showed commercial complementary foods to be consumed by more than 90% of infants aged 6 and 9 months in Europe (13). Yet, little is known about their contribution to nutrient intake, which emphasizes the additional need for data assessing the role of commercial complementary foods.

The Children’s Nutrition Survey to Record Food Consumption (*Kinder-Ernährungsstudie zur Erfassung des Lebensmittelverzehrs*, KiESEL), carried out from 2014 to 2017 (14), addresses the aforementioned data gap. Using the KiESEL data, the objective of the present analysis is to quantify energy and nutrient intakes (including vitamin D supplements) of infants aged ≥ 6 to ≤ 11 months (hereafter referred to as 6–11 months) and to compare the results with the Dietary Reference Values (DRVs) by EFSA (15). Furthermore, the contribution of commercial infant foods to nutrient intakes deviating considerably from DRVs and the impact of vitamin D supplementation is investigated.

## 2 Materials and methods

KiESEL is a cross-sectional study providing food consumption data of infants, toddlers, and preschoolers in Germany (14). The study was conducted by the German Federal Institute for Risk Assessment (*Bundesinstitut für Risikobewertung*, BfR) in 2014–2017 as a module of the German Health Interview and Examination Survey for Children and Adolescents Wave 2 (*Studie zur Gesundheit von Kindern und Jugendlichen in Deutschland Welle 2*, KiGGS Wave 2). KiGGS, in turn, is part of the national health monitoring conducted by the German *Federal Research Institute for Disease Control and Prevention* (*Robert Koch Institute*, RKI) (14, 16). KiESEL received approval by the Berlin Chamber of Physicians (Eth-28/13) and the German Federal Commissioner for Data Protection and Freedom of Information. The primary caregiver for each child enrolled in the study provided written informed consent. To ensure meeting the quality standards in health research reporting, the STROBE-nut guidelines were followed in manuscript preparation (17).

**Figure 1:**
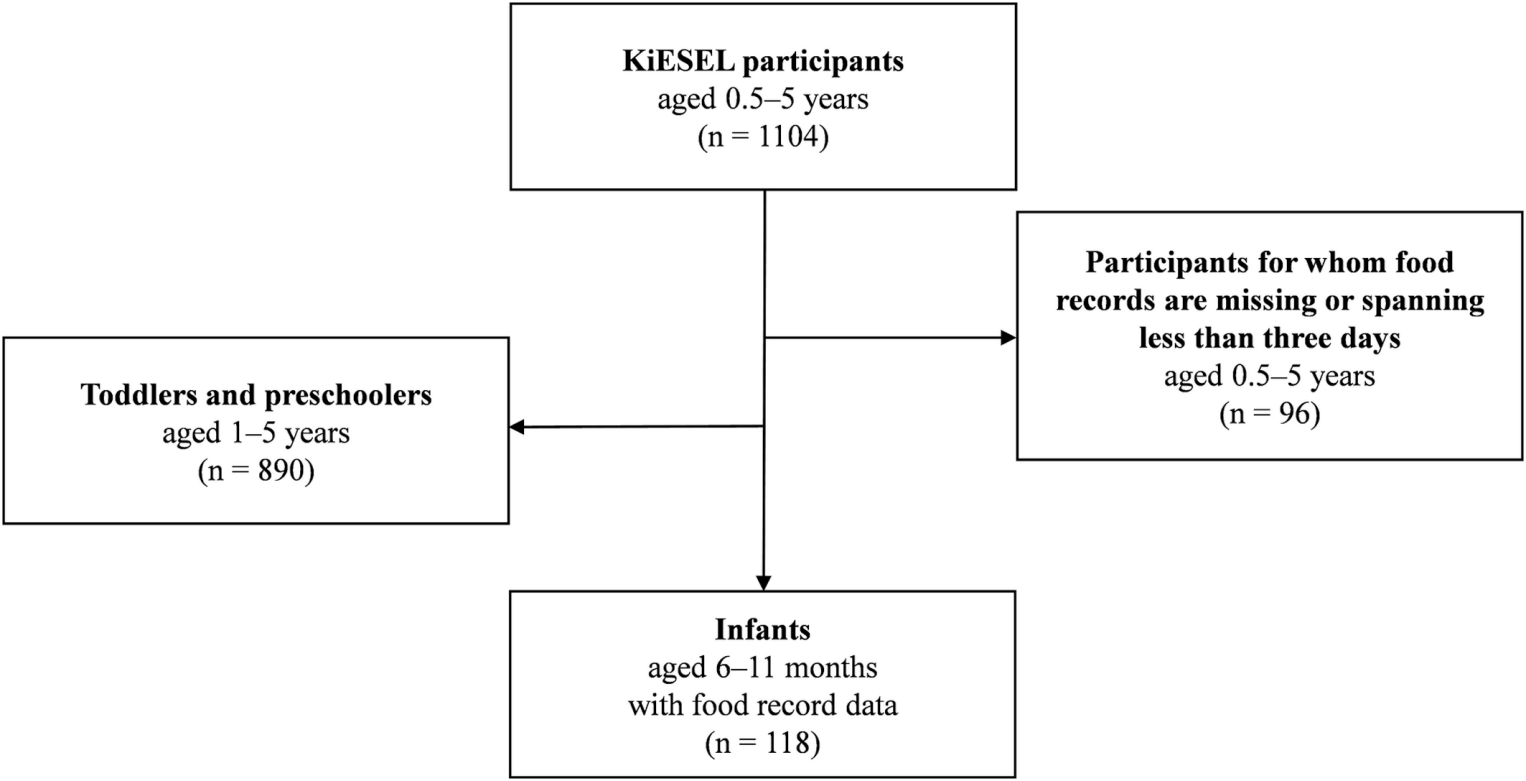
Participant flow chart of the study sample.

The KiESEL participants were randomly selected from the gross sample of KiGGS Wave 2 (18). Sampling in KiGGS Wave 2 was based on official residency registries of 167 representative cities and municipalities initially selected for the KiGGS baseline study (16). The KiESEL sample comprises 1,104 children aged ≥ 0.5 to ≤ 5 years (18). For the present analyses, a subsample of infants 6–11 months of age was formed (*n* = 118) by excluding children aged 1 year and older (*n* = 890) or for whom food records were either missing or spanning less than 3 days (*n* = 96) (Figure 1). Age specifications refer to completed months of life, i.e., the age group “6 months” refers to infants aged 6.0–6.9 months. The child’s age on the interview day, which usually took place 2 days prior to the first food record day, was used as the reference age for all analyses. Further details on the KiESEL study design and survey protocol are described elsewhere (14, 18). For an analysis of nutrient intake in children aged 1–5 years, please refer to Burgard et al. (19).

Data on food and beverage consumption were collected by means of a parent-administered weighed food record encompassing three consecutive days and an additional independent day scheduled 2–8 weeks later (3 + 1 design) (14). Parents received a diary with pre-printed log pages, querying the quantity and specific type (brand, preparation, fat content, etc.) of food and beverages consumed, as well as the time and place of a given eating occasion. During an initial home visit, nutritionists gave personal instructions on how to fill in the weighed food records. For further assistance, kitchen scales (type SOEHNLE Siena) were distributed. Whenever weighing was not feasible, amounts were estimated based on package labels, household measures, or a specifically designed KiESEL picture book. For child day care facilities, a simplified food record was used. In the event of any ambiguities in the protocol entries, the parents were contacted by the study team for clarification (14).

KiESEL data collection further comprised a questionnaire on nutritional behavior and standardized measurements of the child’s weight and length, which were carried out during the home visit (14). Body mass index (BMI) z-scores were calculated following the protocol of the World Health Organization (WHO) Child Growth Standards for children below 5 years of age (20). Infants were classified as having “underweight” or “normal weight”, being “at possible risk for overweight”, or having “overweight/obesity”, if their BMI z-score was < −2 SD, ≥ −2 to ≤ 1 SD, > 1 to ≤ 2 SD, or > 2 SD in relation to the sex- and age-specific reference, respectively (21, 22). To characterize the study population, corresponding sociodemographic data from KiGGS Wave 2 were used, such as the parents’ region of residence and socioeconomic status (SES), the latter capturing the equally weighted dimensions occupation, income, and education (23).

Amounts of human milk were largely estimated based on the frequency of feeding and an age-dependent quantity per feed, i.e., 135 g for infants aged 6–7 months and 100 g for infants aged 8–11 months, as derived by Paul et al. (24). In cases where breastfeeding was reported in the protocol as “throughout the day” (14 protocol entries; *n* = 5 infants) and therefore no feeding frequency was given, an estimate of 600 mL per day (25) was used for analysis from which other amounts of drinking milk (i.e., cow’s milk, infant and follow-on formula) were subtracted. No daily maximum for human milk consumption was defined, as the maximum consumption reported in the protocols was considered plausible.

For the calculation of energy and nutrient intakes, protocol entries were either linked to the German Nutrient Database (*Bundeslebensmittelschlüssel*, BLS) version 3.02 (26) or, in the case of commercial infant foods, to the LEBTAB database (27), taking all details of a food item given in the protocol entry into account (e.g., preparation method, brand) to ensure best possible matching with the food items listed in the databases. In the BLS, nutrient retention factors for vitamins and minerals are considered based on the respective preparation method. LEBTAB is a food composition database covering a wide range of foods explicitly intended for infants and young children, e.g., fortified commercial infant foods. As far as available in the BLS, fortification of other foods, e.g., fruit juices and cereals, was also considered. Vitamin A was calculated as retinol equivalents, vitamin E as *α*-tocopherol equivalents, vitamin K as phylloquinone, niacin as niacin equivalents, and folic acid as folate equivalents.

Supplement use was assessed via a free text box at the end of the food record log pages (18). In a few cases where only the type of supplement, but not the quantity or dosage of nutrients were reported, respective nutrient data were estimated as median from all similar and complete protocol entries, taking the infant’s age into account. The analysis was facilitated by a supplement database, compiled by the BfR (28) and modified by the Max Rubner-Institut (MRI). The term “supplement” refers to both dietary supplements and medicinal products such as vitamin D preparations for rickets prevention. Nutrient intake from supplements was only considered for vitamin D, due to the specific recommendation for routine vitamin D supplementation in infancy. The definition “vitamin D-containing supplement” incorporates all supplements specifying vitamin D as a component, i.e., mono preparations as well as combination preparations with vitamin D and fluoride.

A weighting factor calculated for the total sample of KiESEL children 0.5–5 years was applied to the subsample to compensate for socioeconomic differences compared to the German reference population. This includes sex, age, region, regional structure (e.g., rural areas, large cities), and parents’ education level. The sample weighting accounts for different participation probabilities and corrects deviations of the design-weighted net sample from the German population using population statistics from 2014/2015 and the distribution of educational attainments according to the CASMIN classification (Comparative Analysis of Social Mobility in Industrial Nations) from the Microcensus 2013 (29). Unless stated otherwise, all reported data are weighted.

Analyses were unplanned, and thus, no sample size calculation was carried out. Statistical analyses were performed using SAS version 9.4 (SAS Institute, Inc., Cary, NC, United States). Statistical measures of energy and nutrient intakes of the sample were calculated from individual values, aggregated as arithmetic mean values from all protocol days per child. To account for the skewed distribution of energy and nutrient intakes, medians, 95% confidence interval (CI) of the median and the 10th and 90th percentile (hereafter referred to as P10 and P90) are shown. For metric variables, a difference between groups was considered significant if CIs did not overlap.

Median energy and nutrient intakes were compared to the EFSA DRVs (15). Where available, this work refers to the Population Reference Intake (PRI). In the case of nutrients for which PRIs have not yet been established, the Adequate Intake (AI) was applied. By definition, PRIs and AIs are intended to ensure that the requirements of almost all healthy individuals in a given reference population are met. For this reason, an individual intake below a certain reference value does not necessarily imply an actual deficit, but instead an increased likelihood of inadequate intake. For iron, intakes were additionally compared to the Average Requirement (AR), which refers to the nutrient intake that meets the requirements of 50% of the healthy individuals in a respective population. ARs were further used for assessment of energy intake, considering age- and sex-specific reference values. If applicable, intakes are additionally displayed relative to DRVs (median, interquartile range, minimum-maximum), based on individual intakes as % of the age- and sex-specific DRV.

To identify which food groups are key contributors to the intake of micronutrients with median intakes either below or a multiple above the DRV, the mean percentage contribution to total intake of the selected nutrient is shown for the following five food groups: i. human milk, ii. infant and follow-on formula, iii. commercial processed cereal-based food (e.g., infant cereals), iv. commercial baby food (e.g., ready-to-eat baby food jars, baby tea), and v. ordinary foods and beverages (i.e., all foods not assigned to any of the aforementioned food groups; e.g., homemade complementary foods). The food groups ii.–iv. align with those defined by Regulation (EU) No 609/2013 (30) and are subsequently summarized as commercial infant foods.

## 3 Results

### 3.1 Sample characteristics

Characteristics of the study sample are shown in Table 1. The majority of KiESEL infants were aged ≥ 8 months, had normal weight, and came from families with a medium or high SES. Half of the KiESEL infants received a supplement on at least one of the recorded protocol days, with vitamin D-containing supplements accounting for the vast majority of supplement use.

**Table 1:** Characteristics of KiESEL infants aged 6*–*11 months.^1^

	Total (*n* = 118)	Boys (*n* = 56)	Girls (*n* = 62)
Age (n, %)			
6 months^2^	5 (1.2)	0 (0)	5 (2.2)
7 months	10 (6.1)	7 (10.1)	3 (2.5)
8 months	24 (23.6)	13 (24.8)	11 (22.5)
9 months	28 (26.8)	14 (30.2)	14 (23.8)
10 months	29 (20.7)	11 (16.7)	18 (24.2)
11 months	22 (21.6)	11 (18.1)	11 (24.8)
Anthropometric measurements (mean ± SD)			
Body weight (kg)	8.8 ± 0.9	8.9 ± 0.9	8.7 ± 0.9
Body length (cm)	72.2 ± 3.0	73.3 ± 3.0	71.1 ± 2.7
BMI z-score	−0.09 ± 0.95	−0.45 ± 0.75	0.23 ± 1.03
Weight classification (n, %)^3^			
Underweight (BMI z-score < −2 SD)	3 (1.4)	0 (0)	3 (2.6)
Normal weight (BMI z-score ≥ −2 to ≤ 1 SD)	94 (84.5)	50 (94.8)	44 (75.3)
Possible risk of overweight (BMI z-score > 1 to ≤ 2 SD)	16 (10.4)	4 (4.2)	12 (15.9)
Overweight/obesity (BMI z-score > 2 SD)	5 (3.7)	2 (1.0)	3 (6.2)
Socioeconomic status (n, %)			
Low	5 (9.8)	2 (14.3)	3 (5.7)
Medium	61 (61.6)	28 (60.8)	33 (62.4)
High	52 (28.6)	26 (24.9)	26 (31.9)
Region (n, %)^4^			
North	13 (17.9)	4 (13.7)	9 (21.7)
East	43 (24.6)	18 (19.9)	25 (28.8)
South	36 (28.9)	19 (33.8)	17 (24.5)
West	26 (28.6)	15 (32.6)	11 (25.0)
Supplement use (n, %)^5^			
Any	68 (53.6)	31 (52.8)	37 (54.3)
− Vitamin D-containing	65 (51.7)	31 (52.8)	34 (50.6)

1 Weighted data (n unweighted). SD, standard deviation.

2 Incl. one child 5 months of age.

3 According to the WHO BMI z-score categorization (21, 22).

4 Federal states were assigned as follows. North: Schleswig-Holstein, Hamburg, Lower Saxony, Bremen; East: Berlin, Brandenburg, Mecklenburg-Western Pomerania, Saxony, Saxony-Anhalt, Thuringia; South: Baden-Wuerttemberg, Bavaria; West: North Rhine-Westphalia, Hessia, Rhineland-Palatinate, Saarland.

5 Infants were classified as supplement users if they received supplements at least once during the protocol period.

### 3.2 Energy and macronutrient intake

**Figure 2:**
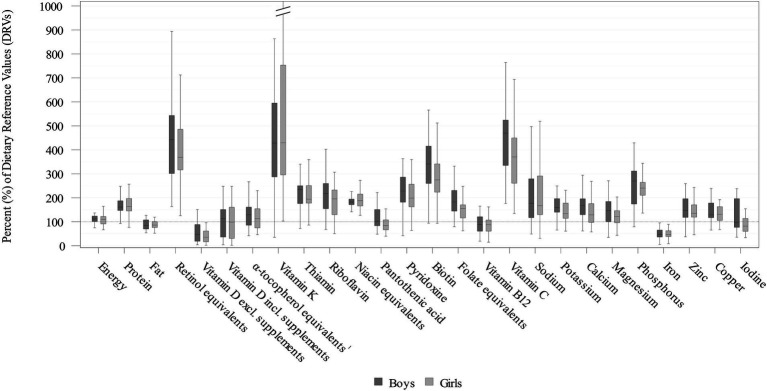
Daily energy and nutrient intakes from food and beverages in infants aged 6–11 months stratified by sex expressed as % of the EFSA DRVs (15) (weighted data; box and whisker plots with median, interquartile range, and minimum-maximum; whisker length limited to 1.5 times the interquartile range, outliers not shown). 51.7% of the KiESEL infants received vitamin D from supplements, therefore total vitamin D intake from food, beverages, and supplements is displayed additionally. ^1^The EFSA AI includes *α*-tocopherol only, while KiESEL intakes are given as α-tocopherol equivalents.

Daily energy and nutrient intakes are provided in Tables 2, 3 and Figure 2 (additionally displaying intakes relative to DRVs). Median energy intake was 10% above the sex- and age-specific ARs (shown in Supplementary Table S1). The percentage of energy intake (E%) provided by fat was about 15% below the AI, while protein intake per kg body weight exceeded the PRI by more than 60% (Table 2; Figure 2; and Supplementary Table S2). With the exception of mono- and disaccharide intake in g/day, CIs did not indicate any significant sex-specific differences in energy and macronutrient intakes.

**Table 2:** Daily energy and macronutrient intake from food and beverages in KiESEL infants aged 6*–*11 months stratified by sex.^1^

	Total (*n* = 118)			Boys (*n* = 56)			Girls (*n* = 62)		
	Median	CI Median	P10, P90	Median	CI Median	P10, P90	Median	CI Median	P10, P90
Energy (kcal)	738	684–784	559, 973	814	715–841	608, 1,262	684	639–739	538, 896
Protein (g)	19.5	17.6–21.1	13.0, 26.9	18.5	16.5–23.2	13.0, 29.5	19.5	17.6–21.1	13.9, 24.5
Protein (E%)	10.4	10.0–10.9	8.3, 12.9	9.5	9.4–10.7	7.5, 11.6	10.9	10.4–11.5	9.4, 12.9
Protein (g/kg body weight)	2.1	2.0–2.3	1.7, 3.1	2.1	1.9–2.3	1.7, 3.3	2.1	2.0–2.6	1.7, 2.7
Fat (g)	27.9	24.8–30.5	19.6, 39.5	30.9	26.1–34.5	20.7, 47.6	24.8	23.7–29.9	19.5, 35.7
Fat (E%)	34.1	33.1–36.7	26.6, 43.7	34.0	31.6–39.5	26.5, 47.9	34.5	31.9–40.1	27.9, 43.5
Saturated fatty acids (g)	10.8	10.3–11.8	7.4, 17.6	10.8	10.4–14.3	7.8, 19.3	10.6	8.9–11.9	6.5, 15.8
Saturated fatty acids (E%)	13.3	12.5–14.3	9.9, 20.8	12.7	12.3–16.0	9.3, 21.1	13.9	12.7–14.7	9.9, 20.3
Monounsaturated fatty acids (g)	9.6	9.0–11.2	6.5, 16.8	11.0	9.1–12.4	6.9, 18.1	9.0	8.6–10.5	6.5, 13.4
Monounsaturated fatty acids (E%)	12.5	11.8–13.5	8.5, 17.6	12.9	11.1–15.1	8.4, 18.3	12.3	11.7–14.2	8.9, 16.4
Polyunsaturated fatty acids (g)	4.6	4.2–4.9	3.2, 7.1	4.9	4.5–5.6	3.7, 7.5	4.2	3.8–4.7	3.1, 6.0
Polyunsaturated fatty acids (E%)	5.6	5.4–6.3	3.9, 8.8	5.4	5.4–5.9	4.3, 9.2	6.2	4.9–6.5	3.9, 7.1
Cholesterol (mg)	43.5	33.3–54.6	6.1, 159.8	37.6	32.4–53.6	2.0, 207.7	51.5	24.5–72.3	8.1, 138.0
Carbohydrates (g)	99.4	90.9–103.1	67.9, 134.5	103.1	99.4–121.4	64.5, 175.2	86.3	77.1–101.5	67.9, 119.9
Carbohydrates (E%)	54.0	51.4–55.5	45.1, 61.2	55.1	50.3–56.6	42.4, 61.3	52.5	49.2–55.8	45.1, 59.3
Mono- and disaccharides (g)*	58.1	53.7–63.1	35.0, 97.8	64.4	58.3–74.8	46.3, 115.2	53.7	47.5–58.1	31.7, 75.1
Mono- and disaccharides (E%)	33.0	31.1–36.0	21.9, 41.3	36.5	32.4–38.7	25.8, 42.3	31.7	29.6–33.1	21.6, 39.2
Dietary fiber (g)	7.1	6.3–8.4	2.4, 12.3	7.8	5.8–9.0	0.1, 12.3	6.7	6.2–8.3	2.9, 11.4

1 Weighted data (n unweighted). The age group “infants” refers to all children aged ≥ 6 to ≤ 11 months of age. Energy and nutrient intakes were calculated using the nutrient databases BLS 3.02 (for ordinary foods and beverages) and LEBTAB (for foods and beverages specifically intended for infants and young children). Due to the display of median values, the sum of protein, fat, and carbohydrate E% does not equal 100%. CI Median, 95% confidence interval of the median; E%, percentage of energy intake; P, percentile.

*Statistically significant difference between sexes (95% confidence intervals of the medians do not overlap).

**Table 3:** Daily micronutrient intake from food and beverages in KiESEL infants aged 6*–*11 months stratified by sex.^1^

		Total (*n* = 118)			Boys (*n* = 56)			Girls (*n* = 62)		
		Median	CI Median	P10, P90	Median	CI Median	P10, P90	Median	CI Median	P10, P90
Vitamins	Retinol equivalents (μg)	969	874–1,105	628, 1,479	1,105	891–1,358	694, 1,364	922	821–1,025	597, 1,588
	Vitamin D excl. supplements (μg)	4.1	2.4–5.0	0.5, 9.5	4.7	2.4–7.8	1.2, 15.1	3.5	2.1–4.7	0.5, 9.0
	Vitamin D incl. supplements (μg)^2^	11.2	7.9–12.8	1.2, 18.6	11.3	7.8–14.3	1.6, 17.2	9.8	4.2–13.7	0.6, 19.2
	α-tocopherol equivalents (mg)	6.3	5.5–7.1	3.2, 9.5	6.5	5.9–7.5	3.7, 9.5	5.6	4.3–7.1	2.7, 10.2
	Vitamin K (μg)	42.9	37.0–46.7	20.7, 86.3	42.8	34.9–53.7	24.7, 82.7	42.9	31.9–56.1	19.0, 94.8
	Thiamin (mg)	0.61	0.56–0.71	0.44, 1.04	0.68	0.63–0.79	0.44, 1.32	0.56	0.53–0.71	0.44, 0.96
	Thiamin (mg/MJ)	0.21	0.19–0.24	0.14, 0.30	0.23	0.20–0.25	0.14, 0.30	0.19	0.18–0.24	0.14, 0.27
	Riboflavin (mg)	0.81	0.69–0.89	0.45, 1.54	0.87	0.69–0.97	0.55, 1.95	0.78	0.65–0.89	0.45, 1.03
	Niacin equivalents (mg)	9.5	8.4–9.9	7.0, 12.6	9.3	7.9–10.5	7.4, 15.4	9.5	8.2–9.9	6.6, 11.4
	Niacin equivalents (mg/MJ)	2.9	2.9–3.1	2.3, 3.6	2.9	2.9–3.1	2.3, 3.5	3.0	2.8–3.3	2.5, 4.0
	Pantothenic acid (mg)	2.7	2.5–3.1	1.8, 6.0	3.1	2.7–3.9	2.1, 6.7	2.5	2.1–3.0	1.7, 4.2
	Pyridoxine (mg)	0.63	0.57–0.74	0.41, 0.94	0.68	0.56–0.81	0.47, 0.99	0.60	0.52–0.67	0.39, 0.89
	Biotin (μg)	18.5	15.7–20.5	12.4, 29.5	20.5	15.7–24.9	13.4, 30.3	16.5	14.7–20.0	12.1, 25.5
	Folate equivalents (μg)	130	117–142	88, 265	146	118–168	98, 288	124	101–131	81, 184
	Vitamin B12 (μg)	1.3	1.1–1.5	0.7, 2.2	1.3	1.0–1.8	0.8, 2.0	1.3	1.1–1.5	0.7, 2.3
	Vitamin C (mg)	80.0	72.0–90.4	50.2, 127.1	94.0	78.5–100.6	55.2, 152.9	74.1	64.2–89.0	38.4, 111.3
Minerals	Sodium (g)	0.33	0.28–0.45	0.16, 0.78	0.35	0.26–0.56	0.16, 0.77	0.33	0.28–0.50	0.16, 0.81
	Potassium (mg)	1,090	1,001–1,313	789, 1,663	1,193	1,090–1,421	644, 1870	1,001	987–1,292	802, 1,421
	Calcium (mg)	397	362–472	241, 752	472	394–544	276, 924	360	310–437	221, 685
	Magnesium (mg)	103	92–117	63, 161	117	96–136	61, 161	97	89–112	71, 143
	Phosphorus (mg)	401	376–428	275, 557	432	378–498	275, 687	385	350–414	272, 525
	Iron (mg)	5.1	4.9–5.7	2.5, 8.3	5.3	4.9–6.6	2.5, 12.1	5.1	4.7–6.1	3.2, 8.0
	Zinc (mg)	4.0	3.7–4.7	2.4, 6.3	4.3	3.7–5.4	2.4, 7.5	3.9	3.7–4.7	2.9, 6.1
	Copper (mg)	0.52	0.50–0.58	0.39, 0.77	0.52	0.49–0.66	0.41, 0.81	0.52	0.46–0.58	0.38, 0.71
	Manganese (mg)	1.1	0.9–1.2	0.3, 2.1	1.0	0.7–1.3	0.1, 2.3	1.1	0.9–1.3	0.4, 2.0
	Iodine (μg)^3^	66.2	57.0–75.5	40.0, 145.7	67.1	62.3–100.9	40.8, 166.7	57.3	51.2–75.5	40.0, 107.9

1 Weighted data (n unweighted). The age group “infants” refers to all children aged ≥ 6 to ≤ 11 months of age. Nutrient intake was calculated using the nutrient databases BLS 3.02 (for ordinary foods and beverages) and LEBTAB (for foods and beverages specifically intended for infants and young children). Statistically significant difference between sexes were identified if 95% confidence intervals of the medians do not overlap. CI Median, 95% confidence interval of the median; P, percentile.

2 51.7% of the KiESEL infants received vitamin D from supplements, therefore total vitamin D intake from food, beverages and supplements is included.

3 Possibly underestimated, as iodized salt is not accounted for in default BLS-recipes.

### 3.3 Micronutrient intake

Median vitamin D intake from food and beverages corresponded to less than half the AI (Figure 2; Table 3; Supplementary Table S2). When considering additional vitamin D intake from supplements, median intake met the AI in boys and almost reached the AI in girls. When looking at the supplement users only, median vitamin D intake from food and supplements was 13.9 μg/day in boys and 13.5 μg/day in girls (data not shown). The supplemented vitamin D dosage per administration was highly uniform with 12.5 μg in median as well as P10 and P90 (data not shown).

For vitamin B12, median intakes corresponded to almost 90% of the AI. Additionally, in girls, median pantothenic acid intake was below the AI. Overall, most median vitamin intakes met or mildly exceeded the DRVs. However, intakes of retinol equivalents, vitamin K, and vitamin C exceeded the DRVs by about four times. There were no significant sex-specific differences for any of the analyzed vitamins.

Median daily mineral intakes were mostly above the respective DRVs. However, median iron intakes in both boys and girls was below half the PRI and did not meet the AR (8 mg/day) either. Furthermore, among girls, median iodine intake was nearly one-fifth below the AI, whereas for boys, it was close to meeting the AI. There were no significant sex-specific differences in mineral intakes.

### 3.4 Dietary sources

**Figure 3:**
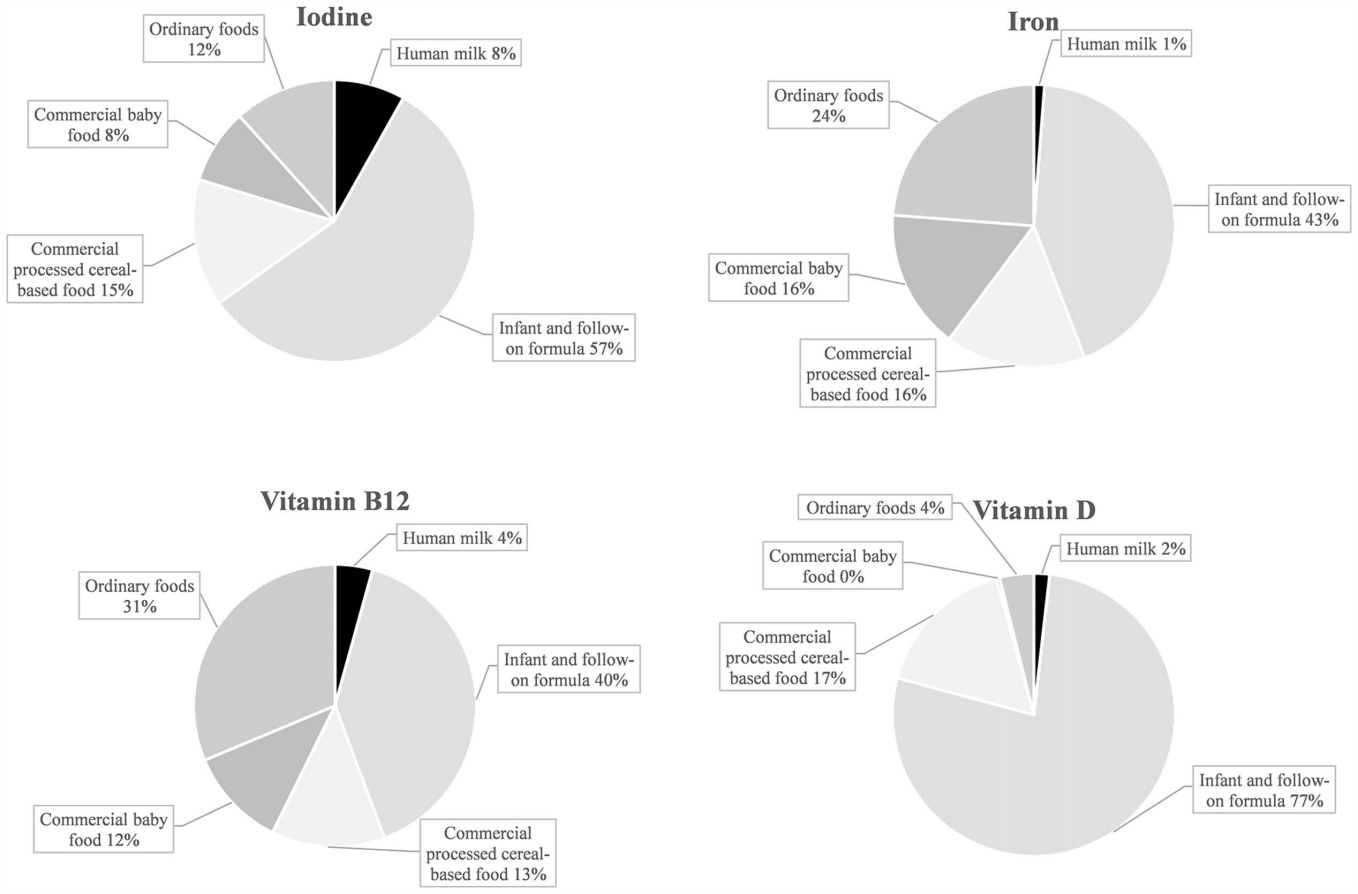
Dietary sources of selected nutrients with median intakes below the EFSA PRI or AI in KiESEL infants aged 6–11 months (expressed as a food group’s mean contribution to total nutrient intake in %).

**Figure 4:**
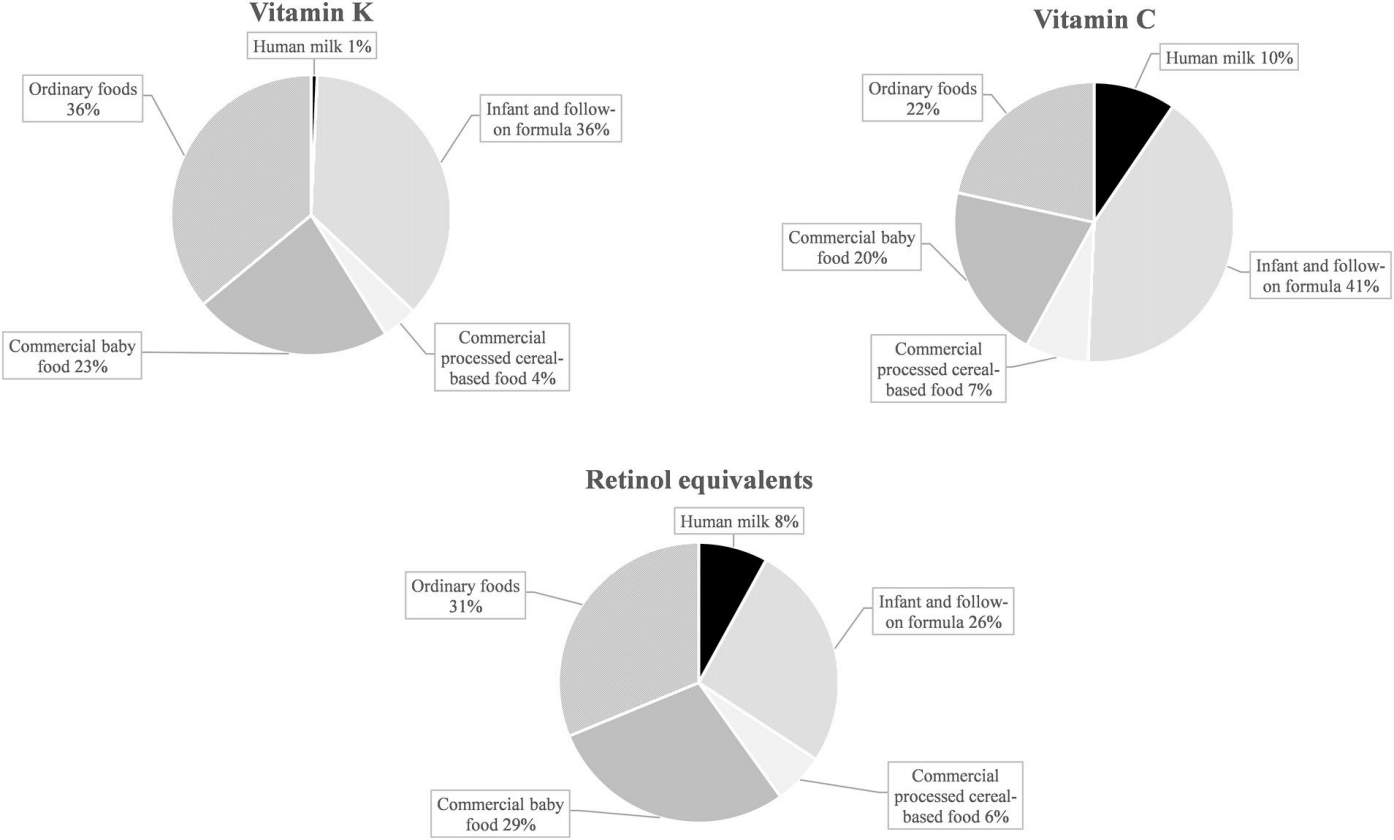
Dietary sources of selected nutrients with median intakes corresponding to about four times the EFSA PRI or AI in KiESEL infants aged 6–11 months (expressed as a food group’s mean contribution to total nutrient intake in %).

Commercial infant foods, particularly infant and follow-on formula, contributed substantially to nutrient intakes. Figure 3 illustrates the dietary sources of micronutrients with median intakes below DRVs: In terms of vitamin D, commercial infant foods were almost the sole dietary sources, accounting for 94% of the overall intake from foods. With 77%, the largest share was accounted for by infant and follow-on formula. For vitamin B12, iron, and iodine, mean contributions of commercial infant foods to nutrient intakes were also high at 65–80% (Figure 3). For retinol equivalents, vitamin K, and vitamin C (all having intakes of a multiple of the DRVs), commercial infant foods contributed 61–68% of the total intake (Figure 4).

## 4 Discussion

Though most nutrient intakes met the DRVs, the present analyses identified iron and vitamin D as critical nutrients among infants aged 6–11 months in a subsample of the representative KiESEL study. The results furthermore indicate potential deficits with regard to iodine in girls and a considerable surpassing of DRVs for retinol equivalents, vitamin K, and vitamin C. Regardless of whether intakes were below or above DRVs, commercial infant foods were found as key dietary sources.

KiESEL infants were found to have a median energy intake mildly above ARs. Yet, the intake is not necessarily to be rated as too high. This may be due to the time lag of at least 2–8 weeks between the interview day decisive for the age group and thus AR assignment and the fourth protocol day, by which the energy requirement may well have increased. It should be further considered that the EFSA ARs are derived from the 2006 growth standards by the World Health Organization (WHO), which slightly differ from the German reference standards, with the latter indicating higher percentiles for all ages (31). As the EFSA ARs potentially underestimate energy requirements, energy intake in KiESEL infants may well be in line with their requirements, underscored by the finding that most infants had a body weight within the normal weight BMI z-score category.

Similar to other European national dietary surveys involving infants (7–9), median protein intake in KiESEL clearly exceeded the PRI, with more than 90% of infants having a protein intake at or above the PRI. While this ensures that protein requirements for healthy growth and development are met, there is growing evidence that high protein intake during early childhood has lasting adverse effects, including increased risk of overweight, obesity, and related diseases later in life (32). On the other hand, following adjustment for potential confounders, a prospective cohort study found boys but not girls with a higher protein intake (comparing tertiles) at the age of 1 year to have lower blood triglyceride concentrations at the age of 6 years (33), suggesting that high protein intake early in life may have both benefits and drawbacks. While EFSA has not yet defined an upper limit for protein intake, the European Society for Paediatric Gastroenterology, Hepatology, and Nutrition (ESPGHAN) Committee on Nutrition recommends that protein intake in complementary feeding should not exceed 15 E% (34). As the P90 for protein E% was below 15 E%, the protein intake observed was within this recommendation for most infants in KiESEL.

Contrary to protein, the DRV for fat was not met by KiESEL infants. This is in accordance with other national dietary surveys reporting median fat intakes in infants in the range of 28–38 E% (6–9). The rationale behind the DRV of 40 E% is to allow for a gradual transition from the high-fat diet provided by exclusive breastfeeding (about 50 E% fat) to the moderate fat intake recommended for older children (30–35 E% fat) (35). Therefore, the AI should be taken as an orientation rather than a strict threshold value, and greater emphasis should be placed on fat quality (e.g., polyunsaturated fatty acids) than quantity. A well-balanced fat composition plays an important role in improving nutrition-related diseases and inflammatory conditions, e.g., by enhancing bioavailability of micronutrients through the lymphatic system (36). For carbohydrate intake, EFSA has not yet established an infant-specific DRV (15).

Consistent with other national dietary surveys (6, 37), median vitamin D intake from foods was well below the AI in KiESEL infants. Inadequate vitamin D status in infancy, leading to low serum calcium concentrations, increases the risk of poor bone mineralization, impaired muscle strength, and, in severe deficiencies, rickets (38), besides potentially rising the risk of infections (39). As a result of limited sun exposure, not least due to rigorous recommendations that infants below 12 months of age should not be exposed to direct sunlight at all (40), cutaneous vitamin D synthesis in infants is restricted. This fact places particular importance on the adequacy of exogenous sources of vitamin D. In line, recommendations on vitamin D intake in infants are usually based on the premise that endogenous vitamin D synthesis is either minimal or non-existent (41). As adequate vitamin D intake requires the use of supplements, national authorities recommend routine vitamin D supplementation of 400–500 international units per day up until a child’s second early summer (39). Yet, the compliance with this recommendation seems low, as only half of the KiESEL infants were reported to be given vitamin D supplements. Although commercial infant foods provided considerable amounts of the total dietary vitamin D intake in KiESEL infants, the AI of 10 μg/day was only reached when intake from supplements was included, confirming the necessity of vitamin D supplementation in infants and promotion of the respective recommendations.

As indicated by median iron intake below the AR of 8 mg/day, more than 50% of KiESEL infants were at risk of insufficient iron intake, confirming iron as a critical nutrient. Iron deficiency may lead to severe and possibly irreversible adverse effects, including impaired cognitive, motor, and behavioral development (42–44). However, iron deficiency cannot be inferred solely from low iron intake but requires the measurement of serum biomarkers, including ferritin. In European comparison, iron intake in KiESEL infants seemed to be in the lower range (6, 9, 37), potentially explained by an up to three times higher meat consumption among infants in the United Kingdom (UK) and Spain (9, 45), and mandatory iron fortification of wheat flour in the UK (46). The major proportion of iron intake in KiESEL infants came from infant and follow-on formula and commercial processed cereal-based food. Correspondingly, Atkins et al. identified formulas and cereals for infants and toddlers as main iron sources in Australian infants (mean age 9 months) (47). In a study conducted in the United States (US), infants aged 6–11 months who were fed infant cereals had higher iron intakes from complementary foods than those not receiving infant cereals (48). As the share of infant cereal consumers in that study was lower among those being breastfed at the time of recruitment compared to those receiving formula, the authors highlighted the need for educating health care providers and caregivers on the importance of iron-rich complementary foods, especially in breastfed infants.

For iodine, a substantial downward deviation from the DRV was seen in girls but not in boys. This is most likely due to differences in the consumption of commercial complementary foods. Due to fortification, these usually contain more iodine compared to homemade complementary foods (49, 50). In KiESEL infants, boys’ mean consumption of commercial processed cereal-based food exceeded that of girls by a third (own unpublished data), resulting in higher iodine intake in boys and thus a sex-specific difference in meeting the DRV. Since iodine intake in infants exclusively receiving homemade complementary foods alongside human milk is likely to be insufficient (49), an iodine supplementation of 50 μg/day is recommended by German authorities for these children (51). In addition, all breastfeeding mothers in Germany are recommended to supplement a daily dose of 100 μg iodine to increase the iodine content in human milk (51). In KiESEL infants, about 80% of the observed iodine intake were provided by commercial infant foods. However, the potentially increased iodine intake from human milk resulting from maternal iodine supplementation is not accounted for in this analysis. In comparison to other European surveys, iodine intake in KiESEL infants was lower (6, 9, 37). A possible explanation is that the consumed amounts of fish (6, 9, 45), dairy (6, 9, 45), and infant and follow-on formula (9, 45) are lower in KiESEL.

There is growing evidence from studies with blood biomarkers such as methylmalonic acid or homocysteine that infantile vitamin B12 deficiency may be more widespread in Europe than previously assumed (52–54). Though the shortfall relative to the AI in KiESEL infants was small, the findings call for further assessment of infantile vitamin B12 status, especially since deficiency can lead to serious consequences, such as delays in psychomotor development (55).

For retinol equivalents, vitamin K, and vitamin C, median intakes corresponded to about a quadruple of the EFSA DRVs, raising concerns that intakes may be too high. While there is no infant specific Tolerable Upper Intake Level (UL) for vitamin A by EFSA, the US Institute of Medicine (IOM) derived a UL of 600 μg/day (56), referring to retinol or retinyl esters. Since the reported intake also includes β-carotene, a regular exceeding of the vitamin A UL in KiESEL infants appears unlikely. Of note, for some infant and follow-on formula products, β-carotene contents of up to 5,000 μg/100 g are listed in the LEBTAB database. This is because infant and follow-on formula commonly contain palm oil, which – according to the BLS data used for nutrient calculation in LEBTAB – is high in β-carotene. However, the value of β-carotene listed in the BLS corresponds to native palm oil. For processed palm oil, as in infant and follow-on formulas, other nutrient databases (e.g., by the United States Department of Agriculture (USDA) (57)) indicate assumed β-carotene levels of 0 μg/100 g or only traces.

For vitamin C, no UL for infants has yet been established by neither EFSA nor IOM. When provisionally using the IOM UL for children aged 1–3 years, set at 400 mg/day (56), excess intake of vitamin C seems unlikely, since the 90th percentiles in KiESEL were far below the UL. Similar to KiESEL, median intakes of vitamin C and retinol equivalents in UK and Spanish infants corresponded to at least three times the EFSA DRVs (9, 37). In Danish infants, similar findings were obtained for vitamin C, but not for retinol equivalents, possibly due to a lower consumption of commercial infant foods (6). For vitamin K, no UL is given by EFSA or IOM, neither for infants nor for young children, and no data on vitamin K intake were found in either of the European national dietary surveys used for comparison.

According to nutrient ranges prescribed by European legislation at the time of the KiESEL study, 100 kcal of ready-to-eat infant and follow-on formula had to provide 24–72% of the EFSA DRV for retinol equivalents, 40–250% of the DRV for vitamin K, and 50–150% of the DRV for vitamin C, respectively (58). Additionally, there are other commercial infant foods that may or must be fortified with vitamin A and vitamin C (50). As the consumption of commercial infant foods was high in KiESEL infants (own unpublished data), this fortification practice explains why intakes of these nutrients tended to be high relative to the DRVs and why large shares of intakes were attributable to commercial infant foods.

Commercial infant foods necessitate being viewed in a differentiated way. In KiESEL infants, they provided considerable amounts of critical nutrients such as iron and iodine and may thus help to ensure nutrient adequacy. At the same time, our results suggest that commercial infant foods might facilitate excess intake of other nutrients, namely retinol equivalents, vitamin K, and vitamin C. Further concerns include early cessation of breastfeeding, predominant sweet taste, higher salt content, lack of ingredient diversity (e.g., bitter vegetables), and limited inclusion of fish (59).

Strengths of the present study are its representative sampling approach along with the use of a weighting factor to adjust the sample to the German population. Furthermore, the use of LEBTAB data ensures brand-specific capturing of commercial infant foods. Since infants with low parental SES are underrepresented and the size of the KiESEL subsample is rather small, the representativity and thus the generalizability of findings is limited. However, a weighting factor was applied to partially offset this limitation. While weighed food records yield highly detailed data, social desirability bias may occur (60) and the associated respondent burden may alter dietary behavior (61). Moreover, the data basis for DRVs in infants is often limited, resulting in DRVs that are frequently based on extrapolations from other age groups (62). Lastly, iodized salt from homemade dishes could only be accounted for if explicitly specified as such, as iodized salt is not accounted for in default BLS-recipes. However, complementary food during the first year of life ought to be free of added salt, rendering the effect negligible.

## 5 Conclusion

The mismatch between certain nutrient intakes and EFSA DRVs in KiESEL infants aged 6 to 11 months necessitates improvement, particularly with regard to iron and vitamin D, and potentially also iodine (primarily in girls). With more than 50% of infants at risk of inadequate iron intake, this finding requires addressing by stakeholders involved in infant feeding counseling and further assessment of iron status in infants in Germany. As only half of the KiESEL infants received vitamin D supplements, the results further highlight the need for increased promotion of the recommendations on vitamin D supplementation in infants aged 6 to 11 months as part of well-child visits. Dietary recommendations and future research on infant nutrient intake ought to differentiate between primary nutrient sources (i.e., predominant feeding of human milk compared to formula, or homemade compared to commercial complementary foods). Finally, the results point to the need for continuous monitoring and adjusting of the fortification levels of commercial infant foods to prevent excess nutrient intake while facilitating the intake of potentially critical nutrients.

## Data Availability

The data analyzed in this study is subject to the following licenses/restrictions: data described in the manuscript, code book, and analytic code will be made available upon request pending application and approval. Requests to access these datasets should be directed to Thorsten Heuer, thorsten.heuer@mri.bund.de.
