# Supplementary material for: Critical vitamin D and iron intakes in infants aged 6–11 months: results from the nationwide German KiESEL study
Source: Front Nutr. 2025 Feb 17;12:1472685. doi: 10.3389/fnut.2025.1472685 (PMC11872716; doi:10.3389/fnut.2025.1472685)
Supplement: Supplementary file 1 [file Table_1.docx]

Supplementary Material

Table S1 EFSA Average Requirements (ARs) for energy (kcal/day) for infants (1)

| **Age** | **ARs (kcal/day)** | |
| --- | --- | --- |
|  | **Boys** | **Girls** |
| 6 months | 597 | 549 |
| 7 months | 636 | 573 |
| 8 months | 661 | 599 |
| 9 months | 688 | 625 |
| 10 months | 725 | 656 |
| 11 months | 742 | 673 |

Table S2 EFSA Dietary Reference Values for macro- and micronutrients for infants (1)^1^

|  | **Dietary Reference Values** | |
| --- | --- | --- |
| **Nutrients** | **Type** | **Value** |
| **Macronutrients** |  |  |
| Water (L/day) | AI | 0.8–1.0 |
| Protein (g/kg body weight)  g/(kg body weight*day) | PRI | 1.31 |
| Fat (E%) | AI | 40 |
| Carbohydrates | Not defined | Not defined |
| **Vitamins** |  |  |
| Retinol equivalents (µg/day) | PRI | 250 |
| Vitamin D (µg/day) | AI | 10 |
| α-tocopherol (mg/day) | AI | 5 |
| Vitamin K^2^ (µg/day) | AI | 10 |
| Thiamin (mg/MJ) | PRI | 0.1 |
| Riboflavin (mg/day) | AI | 0.4 |
| Niacin equivalents (mg/MJ) | PRI | 1.6 |
| Pantothenic acid (mg/day) | AI | 3 |
| Pyridoxine (mg/day) | AI | 0.3 |
| Biotin (µg/day) | AI | 6 |
| Folate equivalents (µg/day) | AI | 80 |
| Vitamin B12 (µg/day) | AI | 1.5 |
| Vitamin C (mg/day) | PRI | 20 |
| **Minerals** |  |  |
| Sodium (g/day) | AI | 0.2 |
| Potassium (mg/day) | AI | 750 |
| Calcium (mg/day) | AI | 280 |
| Magnesium (mg/day) | AI | 80 |
| Phosphorus (mg/day) | AI | 160 |
| Iron (mg/day) | PRI | 11 |
| Zinc (mg/day) | PRI | 2.9 |
| Copper (mg/day) | AI | 0.4 |
| Manganese (mg/day) | AI | 0.02–0.5 |
| Iodine (µg/day) | AI | 70 |
| ^1^ AI, Adequate Intake; E%, percentage of energy intake; PRI, Population Reference Intake.  ^2^ As phylloquinone. | | |

**References**

1. European Food Safety Authority (EFSA). Dietary Reference Values for Nutrients: Summary Report. *EFSA supporting publications* (2017) 14(12):e15121. doi: 10.2903/sp.efsa.2017.e15121.
